# Mechanomyography-Based Metric Scale for Spasticity: A Pilot Descriptive Observational Study

**DOI:** 10.3390/s24165276

**Published:** 2024-08-15

**Authors:** Elgison L. dos Santos, Eduardo M. Scheeren, Guilherme N. Nogueira-Neto, Eddy Krueger, Nathalia Peixoto, Percy Nohama

**Affiliations:** 1 Centro Universitário Internacional Uninter, Curitiba 80020-000, PR, Brazil; elgison.s@uninter.com; 2 Graduate Program in Health Technology, Pontifícia Universidade Católica do Paraná, Curitiba 80215-901, PR, Brazil; eduardo.scheeren@pucpr.br (E.M.S.); nogueira.g@pucpr.br (G.N.N.-N.); 3 Anatomy Department, State University of Londrina, Londrina 86057-970, PR, Brazil; ekrueger@uel.br; 4 Graduate Program in Electrical Engineering, State University of Londrina, Londrina 86057-970, PR, Brazil; 5 Department of Bioengineering, George Mason University, Fairfax, VA 22030, USA; npeixoto@gmu.edu; 6 Graduate Program in Electrical and Computing Engineering, Universidade Tecnológica Federal do Paraná, Curitiba 80230-901, PR, Brazil

**Keywords:** stretch reflex, spasticity evaluation, objective assessment, mechanomyography, biomechanics, Modified Ashworth Scale

## Abstract

(1) Background: The Modified Ashworth Scale (MAS) is commonly used clinically to evaluate spasticity, but its qualitative nature introduces subjectivity. We propose a novel metric scale to quantitatively measure spasticity using mechanomyography (MMG) to mitigate these subjective effects. (2) Methods: The flexor and extensor muscles of knee and elbow joints were assessed with the Modified Ashworth Scale (MAS) during the acquisition of mechanomyography (MMG) data. The median absolute amplitude of the MMG signals was utilized as a key descriptor. An algorithm was developed to normalize the MMG signals to a universal gravitational (G) acceleration scale, aligning them with the limits and range of MAS. (3) Results: We evaluated 34 lower and upper limbs from 22 volunteers (average age 39.91 ± 13.77 years) of both genders. Polynomial regression provided the best fit (R^2^ = 0.987), with negligible differences (mean of 0.001 G) between the MAS and MMG. We established three numerical sets for the median, minimum, and maximum MMG(G) values corresponding to each MAS range, ensuring consistent alignment of the Modified Ashworth levels with our proposed scale. (4) Conclusions: Muscle spasticity can now be quantitatively and semi-automatically evaluated using our algorithm and instrumentation, enhancing the objectivity and reliability of spasticity assessments.

## 1. Introduction

Assessing muscle function is vital for motor rehabilitation. Spasticity, an adverse motor effect affecting individuals with upper motor neuron injuries [1], arises from conditions such as stroke (ST) [2], spinal cord injury (SCI) [3], and cerebral palsy (CP) [4]. It is characterized by a velocity-dependent increase in resistance during passive stretch due to the hyperexcitability of the stretch reflex [5,6].

The Modified Ashworth Scale (MAS) is a widely used clinical tool for assessing spasticity levels [7]. It involves evaluating passive joint movements and grading muscle resistance to stretching from zero (normal tone) to four (severe stiffness) [8]. Despite its popularity, the MAS is subjective and relies heavily on the evaluator’s sensitivity and skill [9,10]. Factors like hyperexcitability, co-contraction of antagonist muscles, and spasms can affect muscle endurance and lead to inconsistent assessments [8]. These subjectivities can result in varying inter-rater evaluations [9], leading to inconsistent analyses, prognoses, and diagnoses. Quantitative data and tools are thus recommended whenever possible.

Quantitative spasticity assessment typically involves biomechanical or neurophysiological approaches [11,12,13] and assessments based on the tonic stretch reflex threshold (TSRT) [14,15,16]. Biomechanical methods often require expensive, complex equipment, making them impractical for routine clinical use. Neurophysiological methods consider neural inputs by measuring alpha and gamma motor neuron excitability, but their clinical relevance remains unclear. TSRT evaluation also needs further validation for repeatability across different evaluators.

Mechanomyography (MMG) has emerged as a method for assessing spasticity. It measures vibrations from muscle contractions and stretching events, recorded on the skin’s surface. MMG sensors are affordable, costing less than USD 100 for a set of 12 sensors that can be easily placed on predefined positions [17]. This non-invasive painless technique is suitable for use in health clinics and outpatient settings for various assessments, including spasticity.

Huang et al. [18] previously explored the relationship between electromyography (EMG), MMG features, and clinical spasticity evaluations. They found a correlation between the MMG and MAS scores (Spearman’s correlation index (ρ) of 0.432, *p* = 0.011). This initial finding spurred further research into MMG, highlighting the need for better algorithms and experimental designs to improve MMG’s clinical reliability. Additionally, it raised the question of whether MAS fully encapsulates spasticity levels or if additional variables could enhance its assessment.

Our team has previously investigated the correlation between MMG signals and MAS-determined spasticity levels [19]. Preliminary findings suggested that the median energy of the MMG signals correlates with the spasticity changes, showing high linear and Spearman correlations with MAS. This indicates the potential for a new metric scale for spasticity assessment using MMG.

Quantifying spasticity with an objective reliable scale enhances the accuracy and safety of assessments, aiding health professionals in diagnosis and treatment decisions. Precise spasticity assessments enable early intervention to prevent contractures and deformities, allowing timely adjustments in medications and therapies based on slight tone variations that subjective assessments might miss. Therefore, this study aims to develop a mathematical model for a new metric scale to assess spasticity, correlating MMG signals with MAS levels. We hypothesized that mechanomyography signals can be accurately correlated with the Modified Ashworth Scale levels. This approach minimizes subjectivity, ensuring consistent monitoring of patient progress, even with different professionals involved in treatment.

## 2. Materials and Methods

### 2.1. Design of Study, Ethics Approval, and Selection of Volunteers

This study was designed as a pilot descriptive observational study, conducted prior to the validation process, and was approved by the Research Ethics Committee of the Federal Technological University of Paraná (UTFPR) (n. 770064). Volunteers were selected from physical therapy patients at a Rehabilitation Hospital Center in Curitiba. The sample was composed by convenience, including all individuals who met the inclusion criteria established by the study, and the recruitment was conducted by the researchers.

The inclusion criteria were patients aged 18 to 70 years with a kinesiological functional diagnosis of either quadriplegia/quadriparesis or hemiplegia/hemiparesis with spastic upper and/or lower limbs. All participants provided informed consent to participate in the study. We did not select for this study patients who had undergone surgery on the spastic limb within six months prior to the study, had used botulinum toxin within three months prior to the study, or had skin lesions at the sensor-mounting site. The total duration of the study was three months.

### 2.2. Data Acquisition

The custom MMG acquisition system used Freescale MMA7260Q triaxial accelerometers, Austin, TX, USA (13 × 18 mm, 0.94 g) with a sensitivity of 800 mV/G (in 1.5 G mode and G = 9.8 m.s^−2^) [12]. For each axis, we verified the oscillation amplitude and frequency using an MTS 647 hydraulic table, lining the axis up with the table’s vibratory base. Control software increased the frequency from 5 to 30 Hz and set the displacement range to 0.5 mm. The table had no certification for frequencies above 30 Hz; so, only frequencies below it were tested [12]. The custom hardware provided 2.2× amplification and a 3rd order 5–50 Hz Butterworth filter. A LabVIEW^®^ virtual instrument, Austin, TX, USA acquired the MMG signals [12] through a National Instruments™NI USB-6221 acquisition, Austin, TX, USA board at a sampling frequency of 1 kHz.

### 2.3. Experimental Setup

**Figure 1:**
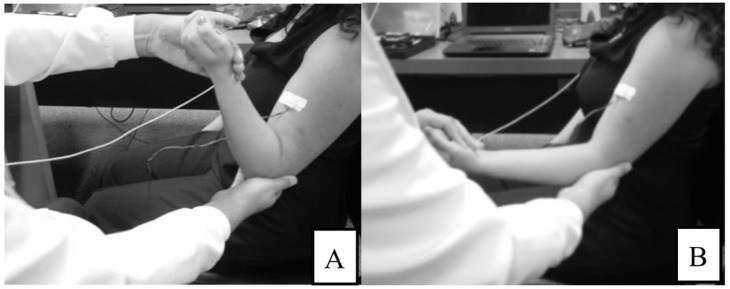
MMG sensor placement on the upper limb and MAS evaluation from elbow flexion (**A**) to extension (**B**).

The room temperature varied between 24.3 °C and 27.3 °C, and the local relative humidity ranged between 53% and 85%. The first step was to prepare the volunteer’s skin with trichotomy and asepsis (70% ethyl alcohol) on the area of sensor fixation. During the upper limb assessments, the volunteer remained seated on a chair with their back supported and left the upper limb free for handling. In the assessment of the lower limbs, the volunteer remained in lateral decubitus on a hospital bed, with the examined lower limb facing up and free to move. Using MAS, a physical therapist assessed the spasticity of the agonist flexor and/or extensor muscles of the knee and/or elbow joints. Figure 1 shows the position of the MMG sensor over the elbow flexor muscle group during MAS evaluation, beginning with elbow flexion (A) and ending with its extension (B). The volunteers were ranked in groups depending on their MAS spasticity level (MAS 0, MAS 1, MAS 1+, MAS 2, MAS 3, and MAS 4).

The MMG signals of agonist and antagonist muscle groups were acquired simultaneously with the MAS evaluation. Triaxial MMG sensors detected vibration signals in three orthogonal directions: perpendicular (Z), transverse (X), and longitudinal (Y) to the muscle fibers. The sensors were fixed to the volunteer’s skin with double-sided tape on the belly of the agonist muscle group.

### 2.4. Signal Processing

A MATLAB (MathWorks^®^, Inc., Natick, MA, USA, v. R2008a) program processed all MMG signals. The analysis windows lasted the whole arc of movement (1–4 s).

The absolute amplitude (Med*_j_*) of the time domain MMG signals was calculated for each *j*th axis using Equation (1).

(1) $${\mathrm{Med}}_{j}=\frac{1}{2}\left(\sqrt{\sum_{i=0}^{n/2-1}{\mathrm{MMG}}_{ji}^{2}}+\sqrt{\sum_{i=n/2}^{n-1}{\mathrm{MMG}}_{ji}^{2}}\right)\;j\in \left\{0,1,2\right\}$$

where

- *n*: the number of samples in a 2 s analysis window;
- *j*: 0 corresponds to X-axis, 1 to Y-axis, and 2 to Z-axis;
- *MMG_ji_*: the *i*-th sample of the *j*-th MMG axis;
- *Med_j_*: average absolute amplitude of the MMG*_j_* axis.

The resultant MMG value (MMG_res_) uses Med*_j_* values in its determination, as stated in Equation (2).

(2) $${\mathrm{MMG}}_{\mathrm{res}}=\sqrt{\sum_{j=0}^{2}{\left({\mathrm{Med}}_{j}\right)}^{2}}$$

### 2.5. A G-Based Scale

The hardware used in signal acquisition exerts direct influence on the obtained data. Most devices express MMG signals in electrical voltage (mV), and this brings inconsistencies that may arise during a multicentric research study. This is because, in general, the MMG equipment varies in location, topology, and technological diversity. A G-based scale is convenient since it avoids problems that originate from using MMG equipment with different gains and accelerometer sensors with different sensitivities. Such a scale can bring studies conducted in different countries onto the same ground.

Two steps are required to transform mV to G, which may work as a common reference, as expressed in Equation (3).

(3) $$|{\mathrm{MMG}}_{\left(G\right)}|=\frac{\mathrm{MMG}}{\mathrm{Gain}\times S}$$

where

- *|MMG_(G)_|*: MMG absolute amplitude (in G);
- *Gain*: the gain of acquisition hardware and/or software;
- *S*: sensor sensitivity.

First, one must determine the hardware (and possibly software) gain (Gain) applied during the acquisition to cancel it out. In this work, only a 2.2× hardware gain was employed. Second, one must adjust the values according to the sensitivity (S) of the MMG sensor employed. In this work, the applied sensitivity was 800 mV/G. Finally, a fitting curve determines an inverse function.

### 2.6. Regression Analyses and Residuals

The statistical analyses were performed with the SPSS Statistics 22.0 software program (IBM Corp., Armonk, NY, USA), and the correlation tests were conducted using MATLAB R2023a (MathWorks, Natick, MA, USA). The ideal mathematical model for composing a quantitative scale based on the MMG(G) signals should distinguish the level of muscular spasticity regardless of the supramedullary pathology assessed (CP or stroke). After data collection, the volunteers were subdivided into two subgroups based on neurological injury: the CP subgroup and the stroke subgroup. Statistical analyses were conducted using the Mann–Whitney test and variance analyses between the subgroups: (1) CP vs. stroke; (2) between male and female genders; and (3) among MAS groups. The results of these statistical tests justified the possibility of creating a single metric scale applicable to both pathologies. If there were no statistically significant differences between the cerebral palsy and stroke subgroups, the MMG signals could be grouped to form a single mathematical model.

**Figure 2:**
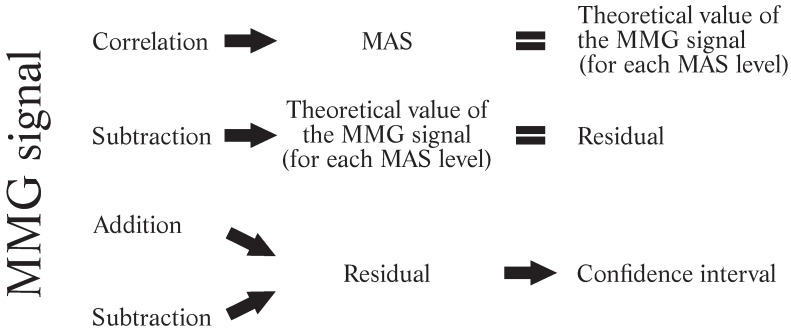
Steps to calculate the residual and confidence interval.

For the conception of mathematical models and the development of a spasticity assessment metric, correlations between MMG(G) signals and MAS groups were performed. Regression analyses confirmed the representativeness of the MMG(G) for muscular spasticity, functioning as an instrument to indicate the spasticity level through the MMG(G) recordings. From the mathematical models generated by these correlations, a theoretical MMG(G) value was obtained for each spasticity level determined by MAS. The residual values were then calculated based on the difference between the actual and theoretical values. Finally, using these residual values, a confidence interval was defined, allowing for a more reliable scale adjustment and determining cutoff points without overlap across different levels. A linear correlation was initially considered because preliminary investigations indicated a high linear correlation between *MMG*(*G*) and MAS [19]. The other regression fitting tested was nonlinear. A second-order polynomial model was tried because it provides a more flexible fitting, consequently producing smaller residuals. The intended model had to classify muscle spasticity regardless of body location. Thus, all spastic muscles of the upper and lower limbs were grouped together during both regression analyses. From the residual values, it was possible to calculate a confidence interval (Figure 2).

## 3. Results

**Figure 3:**
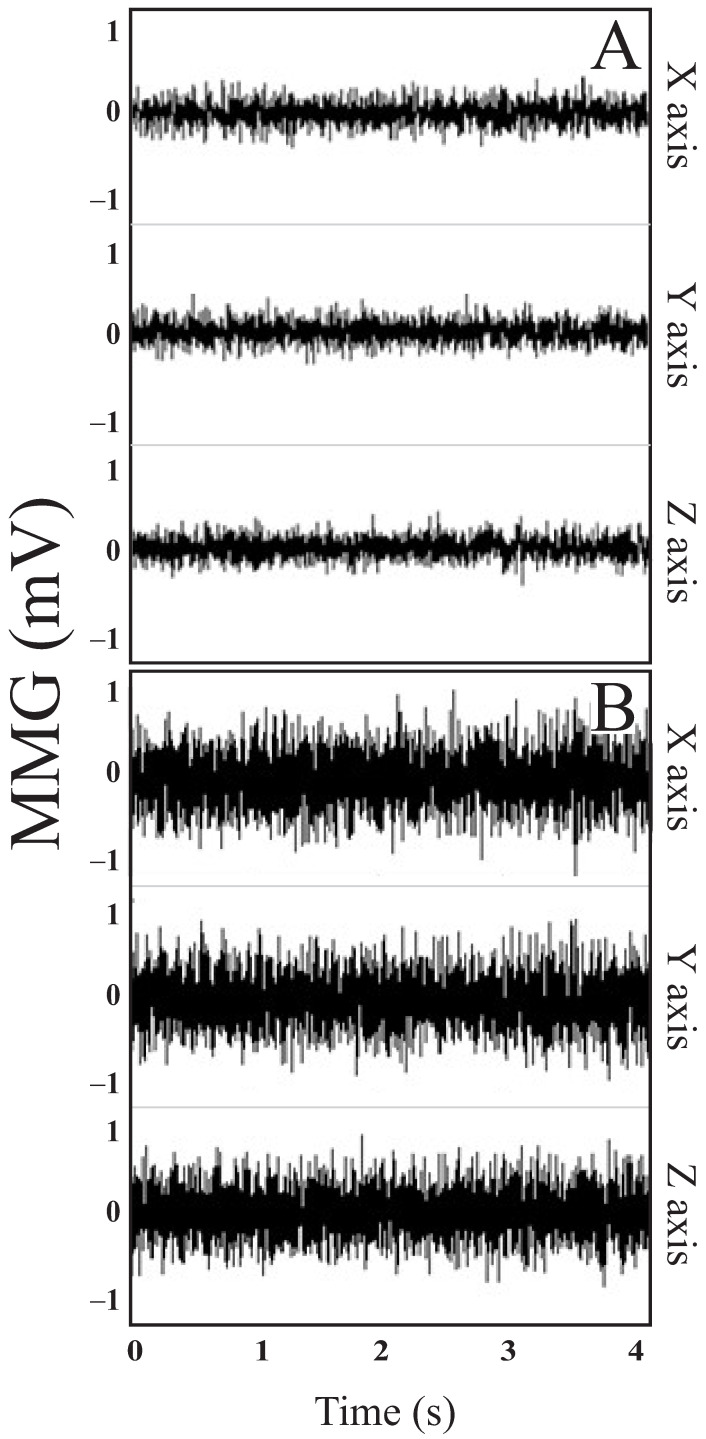
MMG signals (in mV) acquired from a volunteer with MAS 1 (**A**) and MAS 4 (**B**) on the X, Y, and Z axes, respectively.

Our experiments involved 34 limbs from 22 subjects of both genders (11 females and 11 males) with an average age of 39.91 ± 13.77 years. The reported causes of spasticity included stroke (36%), cerebral palsy (18%), traumatic brain injury (18%), spinal cord injury (23%), and amyotrophic lateral sclerosis (5%). The average time since injury was 8.85 ± 10.65 years. Data collection included 38% right upper limb, 23% left upper limb, 18% right lower limb, and 21% left lower limb. About 54% of total body segments had spastic elbow flexors, 11% spastic elbow extensors, 23% spastic knee extensors, and 12% spastic knee flexors. The number of upper and lower limbs in each MAS group was arranged as follows: MAS 0 (6), MAS 1 (6), MAS 1+ (6), MAS 2 (6), MAS 3 (5), and MAS 4 (5). Figure 3A,B shows the raw signals of the three vibration axes (X, Y, and Z) of a representative volunteer of group MAS 1 and 4, respectively.

### 3.1. Mathematical Model Based on Linear Correlation between MAS and $|MMG_{\left(G\right)}|$

**Figure 4:**
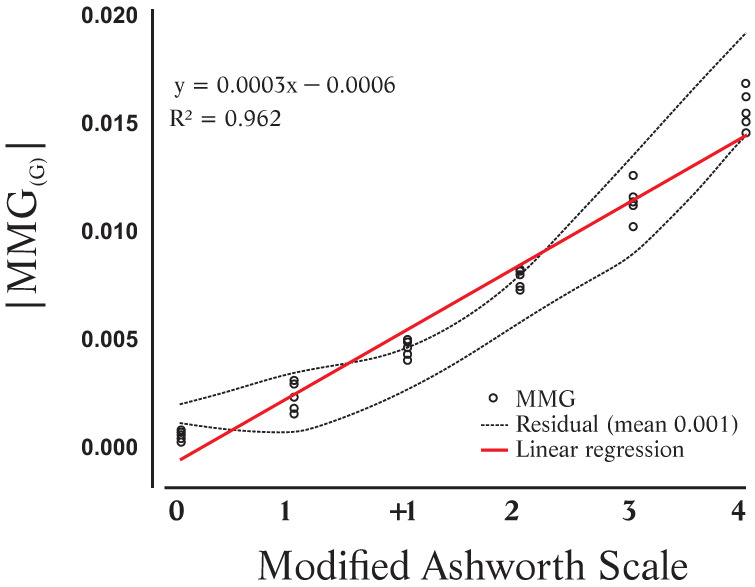
Modified Ashworth Scale and $|MMG_{\left(G\right)}|$ obtained with agonist muscle groups and the residuals.

Figure 4 illustrates the variation in the $|MMG_{\left(G\right)}|$ in relation to the MAS levels with agonist muscle groups of knee and elbow flexors and extensors. Equation (4) defines a simple linear mathematical model in which $|MMG_{\left(G\right)}|$ is the absolute MMG amplitude (in G), and X is the new scale level. Table 1 introduces the proposed values for a new classification of the level of spasticity.

(4) $$X=\frac{|MMG_{\left(G\right)}|\;+\;0.0006}{0.003}$$

### 3.2. Mathematical Model Based on Second Order Polynomial Correlation

**Figure 5:**
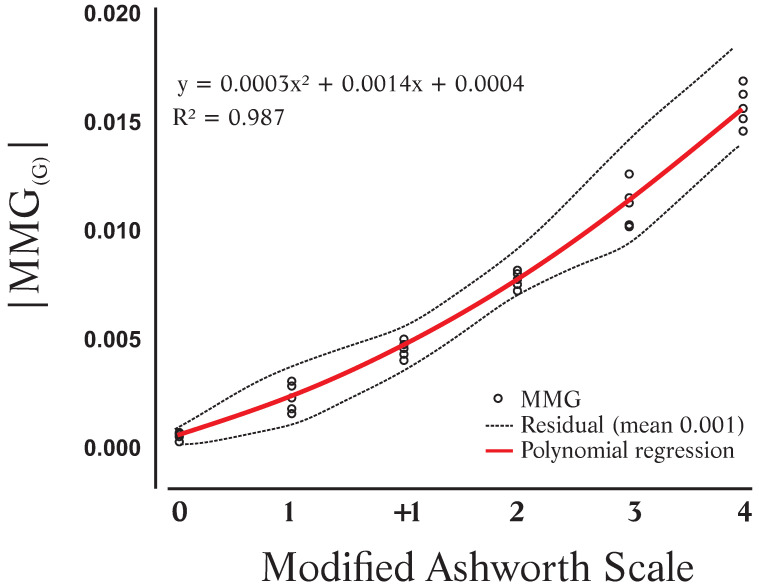
Quadratic correlation between the Modified Ashworth Scale and the $|MMG_{\left(G\right)}|$ for the spastic muscle group (agonist) and the residuals.

Analyzing the response obtained by the quadratic function, this correlation presented a more adequate fit compared to the linear regression, as shown in Figure 5, with the second order polynomial correlation between the MAS and $|MMG_{\left(G\right)}|$ and the residuals.

To consistently lay the basis of the scale, MMG is the independent variable. In spasticity assessment, there are additional requirements: values collected at the same levels of MAS should pertain to the same class of the scale, and $|MMG_{\left(G\right)}|$ values of different MAS levels should pertain to different classes of the new scale. The polynomial correlations between MAS and the median, maximum, and minimum values of $|MMG_{\left(G\right)}|$ allowed the definition of Equations (5)–(7), respectively. These equations delimited the intervals of the second scale, shown in Table 2.

(5) $$|MMG_{\left(G\right)}|\;=10502x^{2}+489.11x-0.0834$$

(6) $$|MMG_{\left(G\right)}|\;=-10438x^{2}+484.83x-0.2597$$

(7) $$|MMG_{\left(G\right)}|\;=-11965x^{2}+510.36x+0.0979$$

## 4. Discussion

This study aimed to develop a mathematical model that could scale the subjective values measured by the MAS using MMG signals, since this scale is the most commonly used for spasticity assessment [3,7]. The findings of our pilot study are promising, with an $R^{2}=0.987$ value for the polynomial fit. Therefore, creating the new scale in correlation with the MAS will be very useful for MAS evaluation, as it will provide quantitative accurate data and allow clinical knowledge to be combined.

Our previous study, which used the MMG to measure spasticity [19,20], indicated that the amplitude of the MMG signal increased significantly in subjects with certain levels of spasticity (e.g., MAS 2) compared to subjects without spasticity (MAS 0). However, the sample in that study was small, and Krueger et al. [20] only considered the linear correlation. The acquired MMG signals only allowed them to distinguish two levels of MAS, since there were insufficient data to propose a new scale. Research proceeded with more volunteers, and the correlation of MMG signals with MAS was examined in both time and spectral domains [19]. Although there was a moderate linear correlation, the median amplitude of MMG signals showed the highest coefficient in time domain analyses. The determination coefficient ($R^{2}$) was 0.962 and 0.893 for the agonist and antagonist muscle groups, respectively. In the spectral analysis, the median frequency indicated differences with the MAS between levels at the extremes of the scale, i.e., between MAS 0 and MAS 4. The result indicated that the highest correlation occurred with the agonist muscle group ($R^{2}=0.488$).

Despite the EMG and MMG having different signal natures (EMG being electrical and MMG being mechanical), the analysis structure for these signals is quite similar. Therefore, techniques used in quantitative spasticity measurement studies with the EMG served as important references for developing our analysis method. For instance, Wang et al. [21] developed an index using the quotient between the $EMG_{rms}$ values of the agonist (biceps brachii) muscle and the sum of the agonist and antagonist (triceps brachii) muscles. They found a significant correlation between the MAS and the EMG-based index for assessing elbow spasticity, but they needed to acquire signals at least in two muscles due to the relationship between agonist muscles. With similar results, our study advanced methodologically by monitoring only one agonist muscle, therefore reducing the practical experimental procedure by at least 50%. Nonetheless, we maintained the strategy of conducting temporal analysis. Consequently, our correlation of the MMG signal in the time domain with the spasticity indices from the MAS aligns with the temporal criteria used by Wang et al. [21]. Our proposal of a Mechanomyography-Based Metric Scale for Spasticity introduces a scale that distinguishes the level of muscular spasticity irrespective of the supramedullary pathology assessed, such as CP or stroke, and across both male and female genders, going beyond previous studies [19,20,21] that analyzed only a specific type of pathology. In this context, our study envisages broader future applications of the method in patient care. Building on this premise, Wang et al. [22] analyzed MMG and EMG signals from the biceps and triceps of post-stroke patients during elbow movements using support vector machine. They evaluated inumerous descriptors from which the RMS, MPF, and MF revealed significant correlation (*p* < 0.05) with the MAS. In contrast to their approach, which used a large amount of data (EMG, MMG, RMS, MPF, and MF), our method focuses exclusively on the MMG signals from the agonist muscle with only one signal descriptor (absolute amplitude), making the application of the method more straightforward for future experiments. This approach enhances the efficiency of data collection and analysis, supporting our aim of effective spasticity assessment.

Figure 4 shows the linear correlation determined by Equation (4). It comprises a set of values between the lowest (MAS 0) and the highest value of $|MMG_{\left(G\right)}|$, obtained (MAS 4). The mean residual is quite high (−0.0641 G). Table 1 has six classes, at equally spaced intervals, which are represented by the metric percentage. With the linear regression, the highest value of MMG was $16.1\times 10^{-2}$ (G). Each class of the proposed scale was normalized as adjusted percentages. However, this division is not consistent, because a greater range of values was involved in the last levels than the others. The last column of Table 1 shows the differences between a metric scale and the one adjusted with the linear regression. The mean difference was 5.93%. For comparison’s sake, the differences obtained with the nonlinear regression were slightly smaller (see Table 2).

The scale in Table 1 led to confusion and did not measure the spasticity correctly. Some $|MMG_{\left(G\right)}|$ values of different MAS levels remained in the same class in the new scale, whereas the $|MMG_{\left(G\right)}|$ of the same MAS level would be in different classes in the new scale. This is a consequence of determining the correlation using only the median values of each MAS group. Therefore, it would not be feasible to maintain a spasticity assessment scale that could raise doubts in evaluators regarding the actual level of involvement. The first attempt in defining a new scale from the linear regression analysis had serious drawbacks. The second-order polynomial correlation presented a more reliable fit between the $|MMG_{\left(G\right)}|$ and MAS. The residuals were smaller (0.001 (G)), and the determination coefficient increased. All proposed classes in this scale were perfectly regular, although there was a minimal deviation. The difference between the metric and the adjusted percentages decreased (mean 3.71%). This correlation showed a better fit in relation to the linear regression line. As seen in Table 2, the new scale did not allow overlapping |MMG(G)| values. The |MMG(G)| values of the same MAS level remained in the same class on the new scale, and |MMG(G)| values of different MAS levels were grouped into different classes. The developed scale does not overlap, because the minimum and maximum values were considered during the adjustment of the scale. Non-overlapping also allows the comparison of the |MMG(G)| values with the MAS levels of spasticity. However, when in doubt about the quantitative scale, classifiers can analyze its result with the corresponding MAS level. In the development of the quantitative scale, the values of two neighbor classes were close to an overlap. The lowest levels of spasticity, precisely the maximum |MMG(G)| value of MAS 0 (without spasticity) and the minimum |MMG(G)| value of MAS 1, were very close. This fact raises doubts in the evaluator due to the subjectivity that MAS allows, and supports the idea that MAS is not effective to determine the level of spasticity.

For the results presented in the correlations, we considered volunteers of different etiologies and different muscle groups. Considering that spasticity might differ depending on whether the injury is either supraspinal or spinal, we did not characterize the categories in this way; at first, the objective was to create an ideal scale for all etiologies, based only on muscle vibration due to movement resistance, regardless of the disease. Thus, to improve the reliability of the scale, further investigations should consider other factors, including the time of injury, the etiology of the lesion, and the anthropometric data. Tissues between muscles and skin (mainly adipose tissue) can alter the features of signals that travel through them, as adipose tissue acts as a low-pass filter and decreases the MMG signal [23]. Considering that the MMG sensors are placed on the surface of the skin, the MMG signals from the levels of spasticity MAS 0 to MAS 1+ could also have significant attenuation and be hard to distinguish since they have small amplitude. Studies are underway to determine the influence of adipose tissue and other anthropometric data. The mass of the sensor also affects the acquired MMG signals. They change with increasing mass, increasing the amplitude and decreasing the median frequency of the signals, the differences became greater when the mass of the sensor changed from 8 to 13 g [24]. In spite of that, there was no indication of a coefficient decrease or increase that could be used in the algorithm of the new scale. Moreover, proposing a scale that only works with a fixed mass of the sensor will make reproducibility impracticable, once biomedical instruments for MMG signal acquisition have sensors of varied dimensions and masses.

## 5. Conclusions

In this study, we developed a mathematical model to automate and quantify spasticity assessment using the MMG. We compared our results obtained from traditional scales and evaluated two quantitative approaches. The linear regression model yielded larger residuals and spasticity levels with irregular intervals. In contrast, the second order polynomial correlation demonstrated the best fit ($R^{2}=0.987$) between the MAS and the MMG-based scale. The residuals were smaller (0.001,(G)), and the spasticity levels were consistent. These findings suggest that a nonlinear mathematical equation can quantitatively measure spasticity using MMG signals. The new metric scale provides quantitative values that minimize intra- and inter-rater errors, enhancing safety during initial diagnosis and therapy follow-up. In conclusion, our proposed scale effectively quantifies spasticity. Furthermore, this semi-automatic method for quantifying MAS classes may facilitate outcome comparisons across different subjects and treatment centers in the future.

We also underscore the importance of considering sensor mass in the final spasticity assessment scale and the need for research to determine the attenuation coefficient to be incorporated into future equations. Consequently, the ideal scale presented may require adjustments to its mathematical models due to various factors affecting the signals, including sensor placement and testing speed. Tests involving intra/inter-test variability, as well as retest reliability, contribute to defining the scale’s reproducibility.

## Figures and Tables

**Table 1:** Quantitative scale for spasticity assessment based on linear regression.

Range $\left(\times 10^{-2}\right)$	Metric/Adjusted (%)	Difference (%)
0.00 $\leq |MMG_{\left(G\right)}|\leq$ 0.90	16.6/5.6	11.0
0.90 < $|MMG_{\left(G\right)}|\leq$ 3.90	33.3/24.2	9.1
3.90 < $|MMG_{\left(G\right)}|\leq$ 6.90	50.0/42.8	7.2
6.90 < $|MMG_{\left(G\right)}|\leq$ 9.90	66.6/61.5	5.1
9.90 < $|MMG_{\left(G\right)}|\leq$ 12.9	83.3/80.1	3.2
12.9 < $|MMG_{\left(G\right)}|\leq$ 16.1	100.0/100.0	0.0

**Table 2:** Quantitative scale for spasticity assessment based on polynomial regression.

Range $\left(\times 10^{-2}\right)$	MAS Level	Metric/Adjusted (%)	Difference (%)
0.0 $\leq |MMG_{\left(G\right)}|\leq$ 0.3	0	16.6/5.9	10.7
0.3 $<|MMG_{\left(G\right)}|\leq$ 1.3	1	33.3/25.5	7.8
1.3 $<|MMG_{\left(G\right)}|\leq$ 2.3	1+	50.0/45.1	4.9
2.3 $<|MMG_{\left(G\right)}|\leq$ 4.4	3	83.3/86.3	−3.0
4.4 $<|MMG_{\left(G\right)}|\leq$ 5.1	4	100.0/100.0	0.0

## Data Availability

Data sharing is not applicable.

## Abbreviations

The following abbreviations are used in this manuscript:

CP	Cerebral Palsy
EMG	Electromyography
$EMG_{rms}$	Root Mean Square of Electromyography
MAS	Modified Ashworth Scale
MF	Median Frequency
MMG	Mechanomyography
MPF	Mean Power Frequency
MVIC	Maximum Voluntary Isometric Contraction
RMS	Root Mean Square
SCI	Spinal Cord Injury
TSRT	Tonic Stretch Reflex Threshold
UTFPR	Federal Technological University of Paraná
